# Expression Analysis of miR-519a-3p and miR-379-5p in Colorectal Cancer Patients: A Combined Experimental and Bioinformatic Approach

**DOI:** 10.3390/diagnostics15162023

**Published:** 2025-08-13

**Authors:** Turkan Gurer, Mehmet Emin Kizakoglu, Alper Aytekin, Rusen Avsar

**Affiliations:** 1 Department of Biology, Faculty of Art and Science, Gaziantep University, Gaziantep 27310, Turkey; byemin2746@gmail.com (M.E.K.); rusen.avsar@gmail.com (R.A.); 2 Department of General Surgery, School of Medicine, Gaziantep University, Gaziantep 27310, Turkey; aytekinalper83@hotmail.com

**Keywords:** colorectal cancer, miR-379-5p, miR-519a-3p, RT-qPCR, bioinformatic analysis

## Abstract

**Background/Objectives:** Colorectal cancer (CRC) is one of the most common malignancies worldwide. microRNAs (miRNAs) are small non-coding RNA molecules that regulate gene expression post-transcriptionally and have emerged as important regulators in cancer biology. This study aimed to investigate the roles of miR-379-5p and miR-519a-3p in CRC using Quantitative Real-Time PCR (RT-qPCR) and comprehensive bioinformatic analyses. **Methods:** Tumor tissues and matched adjacent normal tissues were collected from 54 patients with CRC. The expression levels of miR-379-5p and miR-519a-3p in these tissues were determined using the RT-qPCR method. To investigate the functional roles of differently expressed miRNAs, Gene Ontology (GO) and Kyoto Encyclopedia of Genes and Genomes (KEGG) pathway enrichment analyses were performed to construct miRNA–transcription factor (TF)–target gene–disease interaction networks. **Results:** It was found that the expression level of miR-379-5p was statistically significantly increased in tumor tissues compared to normal tissues, while miR-519a-3p was decreased (*p* < 0.05). GO analysis revealed enrichment in several important biological processes, including cellular protein metabolic processes, biosynthetic processes, response to stress, and nucleic acid binding TF activity. KEGG analysis exhibited that dysregulated miRNAs were associated with important pathways related to carcinogenesis, such as p53 signaling, TGF-beta signaling, and FoxO signaling pathways. Additionally, the miRNAs-TFs-Genes-Diseases Networks analysis identified ESR1 and FOXA1 as common target TFs of dysregulated miRNAs. Network analyses showed that dysregulated miRNAs interact with CRC-associated genes (Caspase 3 (*CASP3*), Adenomatous polyposis coli (*APC*), and AKT serine/threonine kinase 3 (*AKT3*)). **Conclusions:** The present study indicates that miR-379-5p and miR-519a-3p may be involved in CRC progression, with miR-379-5p being upregulated and miR-519a-3p being downregulated in tumor tissues. However, further functional studies are required to clarify their potential roles in tumor biology. The findings of the study suggest that miR-379-5p and miR-519a-3p may be associated with regulatory pathways related to CRC. These miRNAs have the potential to serve as diagnostic biomarkers or therapeutic targets in CRC.

## 1. Introduction

Colorectal cancer (CRC) is a diverse malignancy arising from the colon or rectum and represents one of the leading cancer types globally. Based on 2024 epidemiological data, CRC holds the third position among the most commonly diagnosed cancers in both sexes. It is reported as the third leading cause of cancer-related mortality in men and the fourth in women [1]. CRC formation is a multi-step process driven by a number of genetic and epigenetic processes that lead to the progression from normal mucosal polyps to carcinoma. Together, these events enable cells to bypass many normal regulatory mechanisms, resulting in malignant characteristics such as uncontrolled proliferation, resistance to apoptosis, and tissue invasion [2]. Various environmental and genetic factors contribute to CRC pathogenesis. These include age, sex, alcohol and tobacco use, obesity, dietary habits, physical inactivity, and underlying chronic diseases such as diabetes mellitus, inflammatory bowel disease, and pre-existing colon polyps [3].

microRNAs (miRNAs) are short, single-stranded, non-coding RNA sequences of about 22 nucleotides that play an essential role in modulating gene expression by binding to the 3′ untranslated regions (3′UTRs) of their target transcripts [4,5]. The 3′UTR of a gene can contain multiple binding sites for a wide variety miRNA molecule. Conversely, one miRNA molecule has the capacity to interact with the 3′UTRs of multiple target transcripts. Through this mechanism, numerous miRNAs can finely tune gene expression, and a single miRNA can simultaneously co-regulate several genes [6]. miRNAs are widely distributed across eukaryotic organisms and some of the miRNAs are associated with various types of cancer [7]. miRNAs are involved in many biological pathways associated with cancer, including uncontrolled cell proliferation, angiogenesis, invasion, metastasis, and apoptosis. Many research studies have reported that miRNAs can increase malignant formation by suppressing tumor suppressor genes or increasing oncogene expression and miRNAs can function as clinical markers in various carcinogenesis mechanism [5,8]. Recent studies have demonstrated that the expression levels of miR-2861 and miR-5011-5p are significantly reduced in CRC tissues [9]. miR-564 and miR-718 downregulation in colorectal tumor tissues were associated with tumor suppressor functions [10]. In another study, miR-17-5p was upregulated in CRC tissues compared to adjacent normal tissues [11]. Therefore, miRNAs are increasingly recognized as key regulators of tumor biology.

Multiple investigations have reported that miR-379-5p, mapped to chromosome 14q32.31, exhibits variable expression patterns across a range of malignancies, including endometrial cancer [12], glioma [8], ovarian [13], oral squamous cell carcinoma [14], bladder [15] and hepatocellular carcinoma [16]. miR-519a-3p, which is located on chromosome 14, was dysregulated in various diseases, such as gastric cancer [17], breast cancer [18,19], neuroblastoma [20], Alzheimer’s disease [21], and Parkinson’s disease [22].

As a result of our preliminary study using bioinformatics analysis tools, we determined that some of the genes targeted by miR-379-5p and miR-519a-3p play a role in colorectal carcinogenesis. To the best of our knowledge, the association between miR-379-5p and miR-519a-3p and CRC has not yet been clearly established. Moreover, the specific target genes of miR-379-5p and miR-519a-3p, along with their involvement in signaling pathways and biological processes, remain undefined. Additionally, the target transcription factors (TFs) of miR-379-5p and miR-519a-3p, the genes regulated by these TFs, and their association with related diseases have not been demonstrated. Therefore, the present study aims to investigate the functional roles of miR-379-5p and miR-519a-3p in CRC.

## 2. Materials and Methods

### 2.1. Clinical Specimens

A total of 54 patients with histopathological confirmed CRC (31 males and 23 females; 33 with colon cancer and 21 with rectal cancer) who were admitted to Gaziantep University Hospital between 2017 and 2020 were enrolled in this study. Paired tumor and adjacent non-tumor colorectal tissue specimens were obtained from each patient. Inclusion criteria comprised a confirmed diagnosis of colorectal adenocarcinoma and the absence of any previous chemotherapy or radiotherapy. Exclusion criteria included a history of other malignancies, autoimmune or inflammatory diseases, cardiovascular diseases within the past six months, active infections, long-term immobilization, and inability to undergo surgical intervention. Tissue samples were quickly frozen in liquid nitrogen and stored in RNAlater^®^ solution (Invitrogen; Thermo Fisher Scientific, Inc., Carlsbad, CA, USA) at −80 °C. Ethical approval for this research was granted by the Gaziantep University Ethics Committee, Turkey (Approval No. 2020/114). All participants provided written informed consent prior to inclusion in the study. The research procedures adhered to the ethical standards outlined in the Declaration of Helsinki.

### 2.2. miRNA Isolation from Tissue Samples of CRC Patients

Tissue specimens obtained from CRC patients were preserved in RNAlater^®^ solution and kept at −80 °C. Prior to miRNA extraction, the tissues were thoroughly homogenized. Total RNA, including miRNAs, was extracted from the tumor samples using the mirVana™ miRNA Isolation Kit with phenol (Invitrogen, Thermo Fisher Scientific, Waltham, MA, USA, AM1560) following the manufacturer’s guidelines. RNA concentration and purity were assessed via a NanoDrop spectrophotometer (Maestrogen, Hsinchu, Taiwan), while RNA integrity was confirmed by electrophoresis on a 1% agarose gel. All isolated RNA samples were subsequently stored at −80 °C.

### 2.3. cDNA Synthesis by the Reverse Transcriptase PCR

cDNA was generated using the Reverse Transcriptase PCR (RT-PCR) technique with the TaqMan™ Advanced miRNA cDNA Synthesis Kit (Applied Biosystems, Thermo Fisher Scientific, Waltham, MA, USA, A28007). The resulting cDNA products were preserved at −20 °C until analysis with Quantitative Real-Time PCR (RT-qPCR).

### 2.4. Quantitative Real-Time PCR Assays

The Quantitative Real-time PCR (RT-qPCR) was performed using StepOne & StepOnePlus Real-Time PCR Systems (Applied Biosystems, USA) to assess expression levels of miR-379-5p, miR-519a-3p, and *RNU6B* as housekeeping gene. TaqManTM Advanced miRNA Assay (Applied Biosystems, Thermo Fisher Scientific, Waltham, MA, USA, A25576), TaqManTM Primer Probe (Thermo Fisher Scientific, Waltham, MA, USA) and TaqManTM Fast Advanced Master Mix (Thermo Fisher Scientific, 4444557, Applied Biosystems, USA) were used for RT-qPCR [23]. The sequences of primers are: miR-379-5p: Forward 5′-TGGTAGACTATGGAACGTAGG-3′, Reverse 5′-CGAGGAAGAAGACGGAAGAAT, miR-519a-3p: Forward 5′-AAAGTGCATCCTTTTAGAGTGT-3′, Reverse: 5′-CGAGGAAGAAGACGGAAGAAT-3′; *RNU6B*: Forward 5′-GCTTCGGCAGCACATATACTAAAAT-3′; Revers 5′-CGCTTCACGAATTT GCGTGTCAT-3′. Each sample was analyzed in triplicate. The relative expression levels of miR-379-5p, miR-519a-3p were determined using the 2^−ΔΔCt^ method [24].

### 2.5. Statistical Analysis

All variables were expressed as mean ± standard deviation (SD) and analyzed using SPSS (version 22.0.0.0 SPSS, Inc., Chicago, IL, USA). Differences in miRNA expression between tumor tissues and adjacent non-tumor tissues were assessed by calculating ΔCt for each group and applying the 2^−ΔΔCt^ approach. The normality of numerical variables was evaluated with both the Kolmogorov–Smirnov and Shapiro–Wilk tests. Since Ct values followed a normal distribution, paired *t*-tests were used to compare miR-379-5p and miR-519a-3p expression between tumor and non-tumor samples. For fold change values, which were not normally distributed, the non-parametric Mann–Whitney U test was employed to analyze associations with clinicopathological characteristics. Statistical significance was set at *p* < 0.05.

### 2.6. Gene Ontology (GO) Annotation and Kyoto Encyclopedia of Genes and Genomes (KEGG) Pathway Enrichment Analyses of miR-379-5p, miR-519a-3p

Bioinformatics utilizes computational methods to store, retrieve, and analyze biological data, enabling insights into disease mechanisms and gene regulation. It provides a broad range of techniques, including database construction, gene discovery, and data clustering, which are used in cancer research and the investigation of other diseases [25]. The Kyoto Encyclopedia of Genes and Genomes (KEGG) provides extensive data on gene regulatory pathways, along with a visualization tool. As a result, KEGG has become the main source of information for modeling and simulating biological systems and networks [26]. To explore downstream biological pathways, miRNA signatures were analyzed via the DIANA-miRPath v3.0 online platform using KEGG and GO databases. GO enrichment was assessed across three levels: biological processes (BP), cellular components (CC), and molecular functions (MF). Target genes for the included miRNAs were identified through the TarBase/microT-CDs algorithm and subsequently used for functional enrichment analysis. A threshold of *p* < 0.05 was considered statistically significant.

### 2.7. Construction of Gene Regulatory Networks Involving miR-379-5p, miR-519a-3p, and Transcription Factors (TFs)

To elucidate regulatory interactions between miR-379-5p, miR-519a-3p, and transcription factors (TFs), the study utilized the TransmiR v3.0 database (http://www.cuilab.cn/transmir (accessed on 5 June 2025)), which provides information on experimentally validated regulatory relationships between miRNAs and TFs and between TFs and TFs [27]. To further explore the downstream regulatory effects of TFs, version 2.0 of the TRRUST database (Transcriptional Regulatory Relationships Unravelled by Sentence-based Text-mining) was employed (https://www.grnpedia.org/trrust/ (accessed on 5 June 2025)) [28]. TRRUST contains curated TF–target gene interactions extracted from the literature using a text-mining approach. It also includes associated disease annotations for TFs, which allowed us to map TFs to potential disease phenotypes. Additionally, the functional characterization of TFs was performed using data from The Human Protein Atlas (https://www.proteinatlas.org (accessed on 5 June 2025)) [29], which provides functional annotations based on biological pathways and molecular functions.

## 3. Results

### 3.1. Clinical and Pathological Parameters of Patients with CRC

This retrospective analysis included 54 patients diagnosed with CRC, of whom 23 were women. In 30 tumor specimens, the largest dimension measured ≤6 cm. Based on TNM classification, 29 patients were categorized as Stage I–II and 25 as Stage III–IV. Comprehensive demographic, clinical, and pathological characteristics are summarized in Table 1.

### 3.2. Expression Levels of miR-379-5p and miR-519a-3p

**Figure 1:**
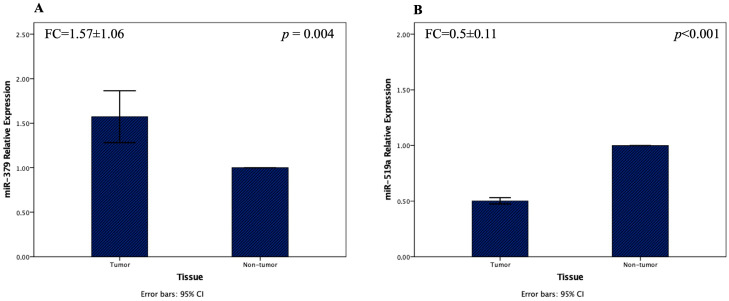
Relative expression of miR-379-5p (**A**) and miR-519a-3p (**B**) in tumor and non-tumor tissues of CRC patients. Expression levels were analyzed by RT-qPCR and normalized using the 2^−ΔΔCt^ method. (Note: Expression levels in normal (non-tumor) tissues were set to 1 as a reference for fold change calculations. Therefore, the bars representing non-tumor tissues appear at a fixed value of 1, and error bars are not visible due to this normalization.) (Data are presented as mean ± standard deviation (SD). Statistical significance was determined using appropriate tests, and *p*-values are indicated.) Note: Expression levels in normal (non-tumor) tissues were set to 1 as a reference for fold change calculations. Therefore, the bars representing non-tumor tissues appear at a fixed value of 1, and error bars are not visible due to this normalization.

Analysis revealed a mean fold change of 1.57 ± 1.06 for miR-379-5p, which was significantly upregulated in tumor tissues compared with adjacent non-tumor samples (*p* = 0.004) (Figure 1A). In contrast, miR-519a-3p exhibited a pronounced downregulation, with a mean fold change of 0.50 ± 0.11 in tumor tissues relative to adjacent controls (*p* < 0.001) (Figure 1B).

### 3.3. The Relationship Between Expressions of miR-379-5p and miR-519a-3p and Clinical-Pathological Characteristics of CRC Patients

The upregulated expression of miR-379-5p was associated with CRC patients age ≥55 years (*p* = 0.031), tumor location in the rectum (*p* = 0.033), advanced clinical TNM stage (Stage III–IV) (*p* = 0.003), and tumor size ≤ 6 cm (*p* = 0.012). However, no statistically significant associations were observed between miR-379-5p expression and gender (*p* = 0.643), perineural invasion (*p* = 0.316), lymphovascular invasion (*p* = 0.708), or histological tumor type (*p* = 0.061). A statistically significant relationship was found between the downregulated expression of miR-519a-3p and the localization of the tumor in the rectum (*p* = 0.01), but no significant association was observed between other characteristics of the patients (*p* > 0.05). The detailed data are presented in Table 2.

### 3.4. GO Annotation and KEGG Pathway Analyses

GO enrichment analysis was performed using the DIANA-miRPath database to functionally characterize the roles of miR-379-5p and miR-519a-3p. The enriched GO terms were categorized under three main domains: Biological Process (BP), Cellular Component (CC), and Molecular Function (MF), as summarized in Table 3. In the BP category, the most significantly enriched terms included: Cellular nitrogen compound metabolic process (GO:0034641), Cellular protein metabolic process (GO:0044267), Biosynthetic process (GO:0009058), Response to stress (GO:0006950). In the CC category, the top enriched terms were: Organelle (GO:0043226), Protein complex (GO:0043234), Cytosol (GO:0005829), Nucleoplasm (GO:0005654). In the MF category, the most enriched terms included: Enzyme binding (GO:0019899), Ion binding (GO:0043167), Nucleic acid binding transcription factor activity (GO:0001071). KEGG pathway enrichment analysis of miRNAs performed using the DIANA-miRPath database revealed significant enrichment of 13 pathways listed in Table 4, including “p53 signaling pathway (hsa04115),” “Endocytosis (hsa04144),” “TGF-beta signaling pathway (hsa04350),” and “FoxO signaling pathway (hsa04068),” which are particularly relevant to colorectal carcinogenesis (Supplementary Table S1).

**Figure 2:**
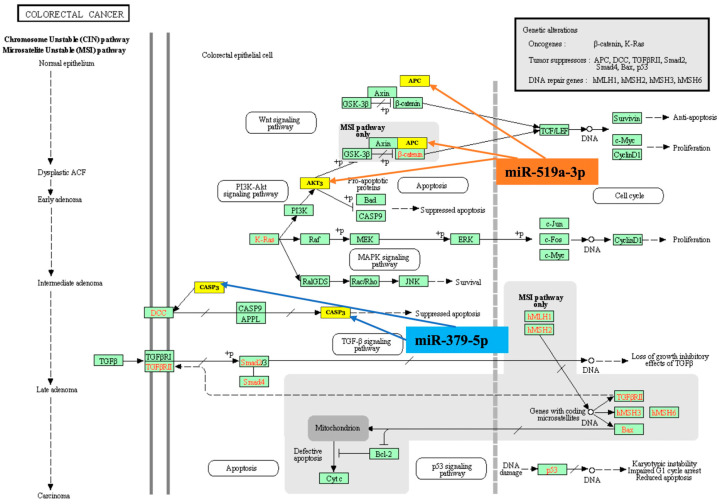
The pathways of target genes of miR-379-5p and miR-519a-3p in CRC in the KEGG (The proteins listed in the yellow box are encoded by genes targeted by either miR-519a-3p or miR-379-5p).

In addition to bioinformatic analyses, the target genes of both miR-379-5p and miR-519a-3p, which play a role in CRC formation, were identified and shown in Figure 2. It was observed that miR-379-5p targets *CASP3*, while miR-519a-3p targets *APC* and *AKT3*. The gene products of *CASP3*, *AKT3*, and *APC* play important roles in the ERK, PI3K, RAS, WNT, and TGFB signaling pathways and apoptosis, which are involved in the development of CRC.

### 3.5. miRNAs-TFs-Genes- Diseases Networks Analysis

**Figure 3:**
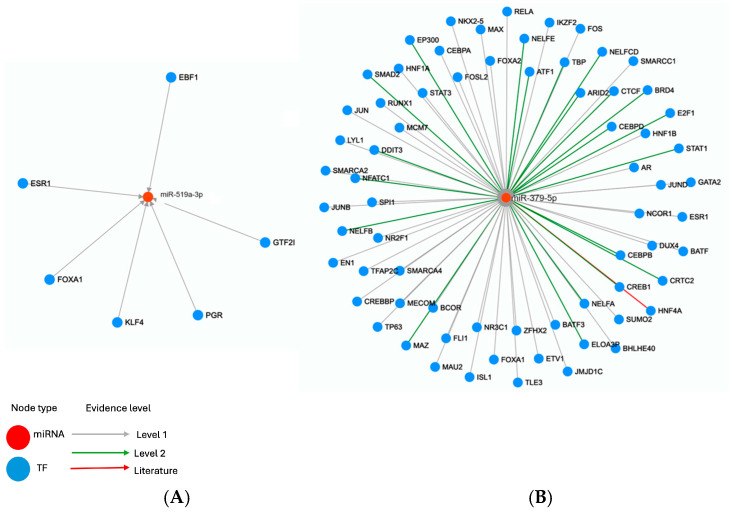
Network of miRNA-mediated regulation of transcription factors (TFs), based on data from TransmiR v2.0, (**A**): miR-519a-3p-TF network and (**B**): miR-379-5p-TF network.

TFs play important roles in regulating the transcription of miRNAs. In the present study, comprehensive regulatory networks between TFs and miR-379-5p and miR-519a-3p were analyzed using the TransmiR v3.0 database. The resulting TF–miRNA regulatory network is illustrated in Figure 3. The analysis revealed that miR-519a-3p interacted with 6 TFs and miR-379-5p interacted with 73 TFs. As a result of the analysis performed using the TransmiR v3.0 online tool, 2 common TFs (Estrogen Receptor 1 (ESR1) and Forkhead box protein A1 (FOXA1)) interacting with both miRNAs were detected. Furthermore, it was determined that miR-519a-3p is effective in the regulation of KLF transcription factor 4 (KLF4) and Progesterone receptor (PGR), while miR-379-5p is effective in the regulation of Signal transducer and activator of transcription 1 (STAT1), SMAD family member 2 (SMAD2), Hepatocyte nuclear factor 4 alpha (HNF4A), MYC associated zinc finger protein (MAZ), Jun protooncogene (JUN), Activating transcription factor 1 (ATF1), and E1A binding protein p300 (EP300).

As TFs play a critical role in the regulation of miRNA expression, and because miRNAs exert their functions by targeting specific genes, TFs interacting with miRNAs, as well as diseases associated with the genes regulated by these TFs, were identified using the TRRUST database. A summary of these findings can be found in Table 5. As shown in Table 5, these TFs were found to function in important biological processes such as proliferation, differentiation, and embryonic development. Additionally, it was revealed that TFs are associated with numerous types of cancer, including CRC [30,31,32,33,34,35,36,37,38,39,40].

## 4. Discussion

CRC represents a significant global public health concern and continues to rank among the most prevalent malignancies worldwide. In recent years, accumulating evidence has demonstrated a strong association between miRNAs and the development and progression of multiple human cancers. These small non-coding RNAs have attracted increasing attention from researchers due to their important roles in cell differentiation, biological development, and the formation and progression of diseases, including cancer. However, the factors affecting miRNAs in this process, and the reasons behind their varying expression in different tumor types, are still not clearly understood. This study compared the expression profiles of miR-379-5p and miR-519a-3p between tumor and adjacent normal tissues in patients with CRC and conducted bioinformatic analyses for these microRNAs. The findings revealed a significant upregulation of miR-379-5p in tumor samples compared to corresponding normal colon or rectal tissues. A review of previous studies indicated that the regulation of miR-379-5p varies with cancer type, showing either downregulation or upregulation. For example, elevated miR-379 expression has been reported to enhance tumor proliferation and facilitate bone metastasis in prostate cancer tissues and cell lines. The same study also found an association between miR-379 expression and the progression-free survival of prostate cancer patients [41]. Unlike the results of our study, several prior investigations have reported that miR-379-5p is downregulated in different cancer types and linked to tumor development. Liang et al. demonstrated that miR-379-5p inhibited the growth, migration, and invasion of endometrial cancer cells [12], while Shukla et al. observed its downregulation in ovarian cancer, both in cell lines and patient-derived tumor specimens [13]. Another study revealed that miR-379-5p was downregulated in serum samples of oral squamous cell carcinoma patients [14]. Moreover, Mosaad et al. discovered that miR-379-5p expression levels was significantly decreased in endometrial cancer tissues by targeted Receptor Tyrosine Kinase Like Orphan Receptor 1 (ROR1) [42]. Zhang et al. showed that miR-379-5p increased the proliferation of articular chondrocytes in osteoarthritis patients by regulating the PI3K/AKT pathway [43]. In addition to these studies in the literature, miR-379-5p was also found to be down-regulated in lung adenocarcinoma [44], glioblastoma [45] and hepatocellular carcinoma [16].

In addition, we analyzed the expression of miR-519a-3p in CRC and observed a reduction in its levels in tumor tissues relative to adjacent normal tissues. Consistent with these findings, Gu et al. reported that miR-519a-3p expression was also suppressed in osteosarcoma cells [46]. Li et al. found down-regulation of miR-519a and up-regulation of its target gene Signal transducer and activator of transcription 3 (*STAT3*) in relapsed glioblastoma tissues compared to tissues from patients with primary glioblastoma. The researchers found a significant inverse correlation between miR-519a and *STAT3* expression levels [47]. Likewise, previous studies have indicated that miR-519a acts as a tumor suppressor in non-small cell lung cancer by regulating *STAT3*, thereby inhibiting tumor advancement [48]. Another study reported that miR-519 expression was significantly reduced in pancreatic cancer cell lines in a hypoxic environment, indicating that miR-519 may play a suppressive role in hypoxia-induced oncogenic phenotypes of pancreatic cancer [49]. In contrast to the results obtained from our study, miR-519a-3p expression level in the serum exosomes of gastric cancer patients with liver metastases was found to be higher than that of gastric cancer patients without metastasis and a correlation was found between high exo-miR-519a-3p expression and poor prognosis [17]. Ward et al. (2014) identified miR-519a as a novel oncomir that regulates the tumor suppressor gene network in breast cancer and leads to resistance to tamoxifen [50]. Moreover, miR-519a-3p has been identified as playing a pivotal role in inhibiting apoptosis of breast cancer cells and reducing their detection by natural killer (NK) cells [18].

In the present study, GO and KEGG pathway analyses were conducted to identify the signaling pathways linked to altered miRNAs, as well as the biological processes, cellular components, and molecular functions related to their target genes. The GO analysis demonstrated that miR-379-5p and miR-519a-3p were associated with 114 genes in biological processes, 113 genes in cellular components, and 115 genes in molecular functions, respectively. KEGG pathway analysis exhibited that dysregulated miRNAs were associated with signaling pathways such as p53, Transforming growth factor beta (TGF-β), and FoxO signaling pathways, which have important functions in carcinogenesis. In support of our findings, many researchers have previously demonstrated that p53, TGF-β and FoxO signaling pathways are effective in CRC formation and progression. An important tumor suppressor, p53 is an important transcription factor that regulates various cellular responses to prevent the transformation of a normal cell into a cancer cell. In the literature, it is reported that p53 mutations are present in 43% of all CRCs [51]. The TGF-β pathway is essential for numerous key biological functions, such as cell differentiation, proliferation, growth, programmed cell death (apoptosis), epithelial–mesenchymal transition (EMT), remodeling of the extracellular matrix (ECM), and angiogenesis. Alterations in TGF-β signaling are effective in many cancer types, including CRC [52]. The Forkhead box (FOX) family of transcription factors has multiple important roles during human development. FOX gene group transcriptional defects in this pathway, which is associated with numerous molecular signaling pathways, have been associated with several types of human cancer. Alterations in at least 14 FOX gene groups have been reported to be associated with CRC formation [53]. In the current study, it was also demonstrated that *CASP3*, *APC*, and *AKT3*, which play important roles in colorectal carcinogenesis, are targeted by miR-379-5p (*CASP3*) and miR-519a-3p (*APC* and *AKT3*) (Figure 2). CASP3, an important mediator of apoptosis, functions as an important component of the cell death mechanism as a result of cells being exposed to cytotoxic drugs and radiotherapy. However, recent studies have also shown that CASP3, which is used as a marker for the efficacy of cancer treatment, also plays non-apoptotic roles such as tumor recurrence and tumor angiogenesis. Zhou et al. reported that CASP3 regulates the migration, invasion, and metastasis of colon cancer cells [54]. It is known that somatic mutations in the *APC* gene play an initiating role in approximately 80% of all CRCs [55]. Another study exhibited a significant increase in the *AKT3* gene in CRC tissues compared to normal tissues [56].

Gene regulation, which has a dynamic and complex structure, is one of the most important mechanisms of biological processes, and disruption of this mechanism can lead to human diseases. Among the molecules that play an important role in regulating gene expression are TFs and miRNAs. The targets of miRNAs include genes that encode TFs. TFs activate or inhibit transcription by binding to specific regulatory sequences in genes. miRNAs can target various TFs and thus regulate TF expression, thereby influencing tumor development [57,58]. In our study, both miR-379-5p and miR-519a-3p were found to have ESR1 and FOXA1 as common target transcription factors (Table 5). Previous studies have shown a relationship between ESR1, which is encoded by the estrogen receptor 1 gene and plays a role in cellular proliferation and differentiation, and CRC [59]. FOXA1, which plays a central role in various biological processes such as organogenesis, differentiation, glycolipid metabolism, proliferation, migration, and invasion, has been reported to be highly expressed in normal human colon tissue but downregulated in colon adenocarcinoma [60]. The information in the literature provides evidence for the relationship between miR-379-5p and miR-519a-3p and CRC. The presence of TFs among the predicted targets of miR-379-5p and miR-519a-3p, many of which are critically involved in essential biological processes such as the regulation of eukaryotic gene expression, cell proliferation and differentiation, embryonic development, intercellular signaling, and chromatin remodeling, further underscores the biological significance of these two miRNAs (Table 5).

The expression of miR-379-5p and miR-519a-3p varies markedly among different malignancies. Although the pathways involved in miRNA synthesis are partly understood and substantial progress has been made in this area, the regulatory mechanisms driving miRNA production and their impact on tumor development remain insufficiently clarified. Recent evidence suggests that miRNA dysregulation can result from gene amplification or deletion, altered transcription factor activity, epigenetic modifications, and changes in genes responsible for miRNA processing. Furthermore, competitive endogenous RNAs (ceRNAs) play an important role in modulating miRNA abundance [61]. In light of the existing data, it is inevitable that the expression levels of the two miRNAs analyzed in our study will either align with or deviate from the findings reported in the literature. Detailed examination of the mechanisms affecting the expression levels of both miR-379-5p and miR-519a-3p in CRC may be the subject of future studies.

Our results suggest that miR-379-5p and miR-519a-3p have potential to serve as biomarkers in CRC. Numerous studies have identified various miRNAs, including miR-21, miR-15b, miR-31 and miR-200c as promising biomarkers for the diagnosis or prognosis of CRC [62,63,64]. It has been demonstrated that these miRNAs are capable of regulating pivotal signaling pathways that are implicated in processes such as cancer proliferation, invasion, and metastasis. The current study indicates that miR-379-5p and miR-519a-3p may also contribute to colorectal carcinogenesis, thus warranting further investigation as potential diagnostic markers. It is important to consider the different expressions and potential functional importance of these miRNAs, as this could enhance the sensitivity and specificity of multi-miRNA panels when combined with already validated miRNAs. Further studies involving larger patient cohorts and functional validation are needed to investigate their additive or synergistic value in CRC biomarker strategies.

In this research, RT-qPCR was used to evaluate miR-379-5p and miR-519a-3p expression in CRC patient tissues, and complementary bioinformatic analyses were conducted to explore their functional significance. Although the study has its strengths, it also has some limitations. Firstly, although the different expressions of miR-379-5p and miR-519a-3p have been clearly demonstrated, the target genes of these miRNAs have not been experimentally investigated. Without directly identifying and validating their targets, it is not entirely accurate to make clear statements about the biological and functional importance of miRNAs based on the changes observed in their expression levels. Future studies that experimentally validate the target genes identified through bioinformatic analyses will be crucial for elucidating the molecular mechanisms by which these miRNAs are involved in CRC initiation and progression. Secondly, the study does not include in vivo and in vitro functional experiments, which limits the ability to determine the direct effects of miR-379-5p and miR-519a-3p on fundamental cellular processes such as proliferation, apoptosis, invasion, and metastasis. In vitro studies using CRC cell lines and in vivo studies using animal models will elucidate the functional roles of these miRNAs and help to validate their potential as diagnostic or therapeutic targets. Ultimately, while this research evaluated the association between miRNA expression profiles and the clinicopathological features of CRC patients, the relatively small cohort size may have restricted both the statistical strength and the broader applicability of the findings. These findings need to be validated in different patient populations and clinical subgroups. In conclusion, while this study provides important preliminary evidence for the dysregulation of miR-379-5p and miR-519a-3p in CRC, further research involving experimental target gene analysis, in vivo and in vitro functional analyses, and larger patient groups is needed to fully understand their roles in the pathogenesis of CRC and their potential clinical significance.

## 5. Conclusions

In this study, a marked increase in miR-379-5p expression and a significant reduction in miR-519a-3p levels were observed in CRC tissues relative to the surrounding normal mucosa in patients with CRC. GO analysis, conducted within the scope of bioinformatic analysis, revealed enrichment in several significant biological processes, including biosynthetic processes, cellular protein metabolic processes, stress response, and nucleic acid-binding transcription factor activity. KEGG analysis demonstrated that dysregulated miRNAs are associated with significant pathways related to colorectal carcinogenesis, including p53 signaling, TGF-beta signaling, and FoxO signaling. Concurrently, the analysis of the network comprising miRNAs, TFs, and genes revealed that ESR1 and FOXA1 are common target TFs of dysregulated miRNAs. Network analyses revealed that dysregulated miRNAs interact with *CASP3*, *APC*, and *AKT3* in CRC formation pathways. As a result, these findings suggest that miR-379-5p expression is increased and miR-519a-3p is decreased in CRC tissues; this may indicate the potential oncogenic and tumor suppressor tendencies of these miRNAs, respectively. However, functional validation analyses are required to determine their specific roles. Findings from this research indicate that miR-379-5p and miR-519a-3p may serve as promising biomarker candidates for CRC. Additional studies are warranted to clarify the molecular pathways involved in CRC development.

## Figures and Tables

**Table 1:** Clinical and pathological parameters of patients with CRC.

*Characteristics*	*Patients n* (%)
**Age (years)**	
≥55	29 (53.7)
˂55	25 (46.3)
**Gender**	
Male	31 (57.4)
Female	23 (42.6)
**Cigarette smoking**	
Yes	15 (27.8)
No	39 (72.2)
**Alcohol drinking**	
Yes	5 (9.3)
No	49 (90.7)
**Tumor location**	
Colon	33 (61.1)
Rectum	21 (38.9)
**Neural invasion**	
Yes	10 (18.5)
No	44 (81.5)
**Lymphovascular invasion**	
Yes	14 (25.9)
No	40 (74.1)
**TNM stage**	
I–II	29 (53.7)
III–IV	25 (46.3)
**Tumor size (cm)**	
≤6	30 (55.6)
>6	24 (44.4)
**Tumor histological type**	
Adenocarcinoma	38 (70.4)
Mucinous adenocarcinoma	16 (29.6)

**Table 2:** The relationship between the clinical and pathological characteristics of CRC patients and the expression of miR-379-5p and miR-519a-3p.

Variables	miR-379-5p Fold Change			miR-519a-3p Fold Change		
	Mean ± SD	*n* (Mean Rank)	*p*	Mean ± SD	*n* (Mean Rank)	*p*
**Age (years)**						
≥55	1.85 ± 1.25	29 (31.79)	**0.031**	0.49 ± 0.77	29 (27.52)	0.993
˂55	1.25 ± 0.71	25 (22.52)		0.52 ± 0.13	25 (27.48)	
**Gender**						
Male	1.62 ± 1.16	31 (28.35)	0.643	0.51 ± 0.12	31 (28.77)	0.490
Female	1.5 ± 0.95	23 (26.35)		0.49 ± 0.09	23 (25.78)	
**Cigarette smoking**						
Yes	1.95 ± 1.51	15 (31.20)	0.284	0.5 ± 0.11	15 (29.40)	0.582
No	1.43 ± 0.82	39 (26.08)		0.5 ± 0.1	39 (26.77)	
**Alcohol drinking**						
Yes	1.9 ± 1.38	5 (28.60)	0.664	0.52 ± 0.14	5 (30.60)	0.885
No	1.54 ± 1.05	64 (27.39)		0.5 ± 0.1	49 (27.18)	
**Tumor location**						
Colon	1.34 ± 0.76	33 (23.85)	**0.033**	0.53 ± 0.13	33 (31.88)	**0.01**
Rectum	1.95 ± 1.37	21 (33.24)		0.46 ± 0.02	21 (20.62)	
**Neural invasion**						
Yes	1.64 ± 0.7	10 (32.00)	0.316	0.5 ± 0.97	10 (27.70)	0.964
No	1.56 ± 1.14	44 (26.48)		0.5 ± 0.1	44 (27.45)	
**Lymphovascular invasion**						
Yes	1.35 ± 0.36	14 (28.86)	0.708	0.48 ± 0.08	14 (23.36)	0.252
No	1.65 ± 1.22	40 (27.03)		0.51 ± 0.11	44 (28.95)	
**TNM stage**						
I-II	1.3 ± 0.85	29 (21.59)	**0.003**	0.5 ± 0.11	29 (27.31)	0.924
III-IV	1.9 ± 1.21	25 (34.36)		0.5 ± 0.1	25 (27.72)	
**Tumor size (cm)**						
≤6	1.8 ± 1.19	30 (32.30)	**0.012**	0.51 ± 0.11	30 (28.00)	0.794
>6	1.29 ± 0.84	24 (21.50)		0.49 ± 0.09	24 (26.88)	
**Tumor histological type**						
Adenocarcinoma	1.7 ± 1.13	38 (30.11)	0.061	0.51 ± 0.12	38 (28.42)	0.507
Mucinous adenocarcinoma	1.28 ± 0.87	16 (21.31)		0.47 ± 0.02	16 (25.31)	

**Table 3:** GO enrichment annotations of miR-379-5p and miR-519a-3p (**A**): Biological Process (BP), (**B**): Cellular Component (CC), (**C**): Molecular Function (MF).

**A**				
**GO Category**	**GO ID**	**miRNAs**	**Target Genes Count**	***p* Value**
Cellular nitrogen compound metabolic process	0034641	miR-519a-3p	17	3.67563626886 ×10^−8^
		miR-379-5p	33	
Cellular protein metabolic process	0044267	miR-519a-3p	1	7.5887688796 × 10^−5^
		miR-379-5p	10	
Biosynthetic process	0009058	miR-519a-3p	14	7.5887688796 × 10^−5^
		miR-379-5p	26	
Response to stress	0006950	miR-519a-3p	10	9.79296263689 × 10^−5^
		miR-379-5p	19	
Symbiosis, encompassing mutualism through parasitism	0044403	miR-519a-3p	3	0.000196157014163
		miR-379-5p	9	
Viral process	0016032	miR-519a-3p	3	0.000251538030074
		miR-379-5p	8	
Gene expression	0010467	miR-519a-3p	3	0.000251538030074
		miR-379-5p	9	
Macromolecular complex assembly	0065003	miR-519a-3p	5	0.000251538030074
		miR-379-5p	10	
Neurotrophin TRK receptor signaling pathway	0048011	miR-519a-3p	4	0.000583602418168
		miR-379-5p	3	
Protein complex assembly	0006461	miR-519a-3p	4	0.00129729179284
		miR-379-5p	9	
Cellular component assembly	0022607	miR-519a-3p	7	0.00201815811768
		miR-379-5p	10	
Fc-epsilon receptor signaling pathway	0038095	miR-519a-3p	3	0.00363189359633
		miR-379-5p	2	
Catabolic process	0009056	miR-519a-3p	7	0.00363189359633
		miR-379-5p	15	
Fibroblast growth factor receptor signaling pathway	0008543	miR-519a-3p	4	0.00417090394179
		miR-379-5p	2	
Positive regulation of nuclear-transcribed mRNA catabolic process, deadenylation-dependent decay	1900153	miR-519a-3p	1	0.00434127923468
		miR-379-5p	2	
Epidermal growth factor receptor signaling pathway	0007173	miR-519a-3p	4	0.00434127923468
		miR-379-5p	2	
Phosphatidylinositol-mediated signaling	0048015	miR-519a-3p	3	0.00605246071122
		miR-379-5p	2	
Positive regulation of nuclear-transcribed mRNA poly(A) tail shortening	0060213	miR-519a-3p	1	0.00744380742909
		miR-379-5p	2	
Blood coagulation	0007596	miR-519a-3p	1	0.00744380742909
		miR-379-5p	7	
Aging	0007568	miR-519a-3p	3	0.00837634698204
		miR-379-5p	4	
Cellular protein modification process	0006464	miR-519a-3p	9	0.00880145157714
		miR-379-5p	13	
Clathrin coat disassembly	0072318	miR-519a-3p	1	0.00998188876802
		miR-379-5p	2	
Vesicle-mediated transport	0016192	miR-519a-3p	7	0.0106960983567
		miR-379-5p	8	
Histone demethylation	0016577	miR-519a-3p	1	0.0124862578037
		miR-379-5p	1	
Extracellular matrix disassembly	0022617	miR-519a-3p	1	0.0124862578037
		miR-379-5p	3	
COPI coating of Golgi vesicle	0048205	miR-519a-3p	1	0.0161387605594
		miR-379-5p	1	
Organ regeneration	0031100	miR-519a-3p	1	0.0206539512892
		miR-379-5p	3	
Angiotensin maturation	0002003	miR-379-5p	2	0.0215777282565
Negative regulation of cyclin-dependent protein serine/threonine kinase activity	0045736	miR-519a-3p	2	0.0215777282565
		miR-379-5p	1	
Cellular response to DNA damage stimulus	0006974	miR-519a-3p	3	0.02427309224
		miR-379-5p	5	
Extracellular matrix organization	0030198	miR-519a-3p	1	0.0246965891574
		miR-379-5p	6	
Nucleobase-containing compound catabolic process	0034655	miR-519a-3p	4	0.0246965891574
		miR-379-5p	8	
Cell–substrate junction assembly	0007044	miR-519a-3p	1	0.0249599495373
		miR-379-5p	1	
Axon guidance	0007411	miR-519a-3p	5	0.0249599495373
		miR-379-5p	4	
Platelet degranulation	0002576	miR-519a-3p	1	0.0270931622974
		miR-379-5p	2	
Regulation of cell cycle	0051726	miR-519a-3p	4	0.0350158897279
		miR-379-5p	3	
Androgen receptor signaling pathway	0030521	miR-519a-3p	3	0.0356378038063
Regulation of gene silencing	0060968	miR-379-5p	2	0.0378283730158
Female pregnancy	0007565	miR-379-5p	4	0.0378283730158
Mitotic cell cycle	0000278	miR-519a-3p	2	0.0378283730158
		miR-379-5p	4	
Response to organonitrogen compound	0010243	miR-519a-3p	1	0.0414573363362
		miR-379-5p	2	
G2/M transition of mitotic cell cycle	0000086	miR-519a-3p	2	0.0414573363362
		miR-379-5p	2	
Prostate gland growth	0060736	miR-519a-3p	2	0.0485118349745
Biological process (GO:0008150): 114 Target Genes				3.09925838529 × 10^−5^
**B**				
**GO Category**	**GO ID**	**miRNAs**	**Target Genes Count**	***p* Value**
Organelle	0043226	miR-519a-3p	32	2.83862016795 × 10^−14^
		miR-379-5p	54	
Protein complex	0043234	miR-519a-3p	15	0.000439974683109
		miR-379-5p	23	
Cytosol	0005829	miR-519a-3p	14	0.000762521273454
		miR-379-5p	16	
Nucleoplasm	0005654	miR-519a-3p	4	0.0182290609436
		miR-379-5p	10	
Male pronucleus	0001940	miR-379-5p	2	0.0286881480324
Prp19 complex	0000974	miR-519a-3p	1	0.0286881480324
		miR-379-5p	2	
Cytoplasmic side of endoplasmic reticulum membrane	0098554	miR-519a-3p	1	0.0405314868526
Female pronucleus	0001939	miR-379-5p	2	0.0405314868526
Protein phosphatase type 1 complex	0000164	miR-519a-3p	1	0.0405314868526
		miR-379-5p	1	
Cellular component (GO:0005575): 113 Target Genes				0.000439974683109
**C**				
**GO Category**	**GO ID**	**miRNAs**	**Target Genes Count**	***p*** **Value**
Enzyme binding	0019899	miR-519a-3p	*8*	0.00284609917602
		miR-379-5p	*10*	
Ion binding	0043167	miR-519a-3p	*21*	0.00284609917602
		miR-379-5p	*26*	
Nucleic acid binding transcription factor activity	0001071	miR-519a-3p	*6*	0.0124666471659
		miR-379-5p	*7*	
Enzyme regulator activity	0030234	miR-519a-3p	*6*	0.0393518807246
		miR-379-5p	*5*	
Molecular function (GO:0003674): 115 Target Genes				1.53107464023 × 10^−5^

**Table 4:** KEGG pathway enrichment analysis of dysregulated miR-379-5p and miR-519a-3p in CRC patients’ tissues.

KEGG Pathways	miRNAs	Target Genes	*p* Value
Endocytosis (hsa04144)	miR-519a-3p	*ARAP2*, *PDGFRA*, *DAB2*, *NEDD4L*, *EEA1*, *PSD*, *PLD1*, *GBF1*, *ZFYVE9*, *HSPA8*, *RAB22A*, *LDLR*, *SMAD7*, *IQSEC2*, *TGFBR2*, *RAB5B*, *ERBB4*	0.000791081026484
	miR-379-5p	*TGFBR1*, *HLA-E*, *EHD4*, *SMAP1*, *DNM3*, *MDM2*	
Gap junction (hsa04540)	miR-519a-3p	*PDGFRA*, *GRM5*, *ITPR1*, *SOS1*, *GUCY1A2*, *PDGFD*, *GJA1*, *MAP3K2*, *MAPK1*	0.00232477710776
	miR-379-5p	*MAP3K2*	
Signaling pathways regulating pluripotency of stem cells (hsa04550)	miR-519a-3p	*STAT3*, *REST*, *FZD6*, *WNT2B*, *INHBA*, *ZFHX3*, *FZD3*, *PCGF5*, *RIF1*, *SMAD5*, *ACVR1C*, *IGF1*, *NEUROG1*, *MAPK1*, *JAK1*, *BMPR2*, *COMMD3-BMI1*	0.00232477710776
	miR-379-5p	*WNT2B*, *PCGF5*	
p53 signaling pathway (hsa04115)	miR-519a-3p	*CDK2*, *RCHY1*, *MDM4*, *IGF1*, *CASP8*, *CDKN1A*, *SESN3*, *PTEN*, *CCNG2*	0.00241402518873
	miR-379-5p	*CCNB1*, *RFWD2*, *MDM2*	
Proteoglycans in cancer (hsa05205)	miR-519a-3p	*ESR1*, *STAT3*, *ROCK2*, *FRS2*, *FZD6*, *TLR4*, *FZD3*, *MMP2*, *HIF1A*, *ITPR1*, *SOS1*, *DDX5*, *IGF1*, *GAB1*, *CDKN1A*, *MAPK1*, *ERBB4*	0.00241402518873
	miR-379-5p	*HBEGF*, *WNT2B*, *PTK2*, *MDM2*	
Hepatitis B (hsa05161)	miR-519a-3p	*STAT3*, *E2F1*, *E2F2*, *CREB5*, *CDK2*, *MAP3K1*, *TLR4*, *CREB1*, *MAPK8*, *CASP8*, *CDKN1A*, *PTEN*, *MAPK1*, *JAK1*	0.00298589620707
	miR-379-5p	*TGFBR1*, *MAP3K1*	
Axon guidance (hsa04360)	miR-519a-3p	*SEMA5A*, *EPHA5*, *ROCK2*, *PPP3R1*, *SRGAP1*, *NTNG1*, *CXCL12*, *PPP3CA*, *DPYSL5*, *DPYSL2*, *NTN4*, *SEMA7A*, *EPHA4*, *MAPK1*	0.00515681863
	miR-379-5p	*ABLIM3*, *PTK2*, *UNC5D*	
Estrogen signaling pathway (hsa04915)	miR-519a-3p	*ESR1*, *CREB5*, *CREB1*, *MMP2*, *ITPR1*, *SOS1*, *HSPA8*, *MAPK1*	0.00939898047994
	miR-379-5p	*HBEGF*	
Glioma (hsa05214)	miR-519a-3p	*PDGFRA*, *E2F1*, *E2F2*, *SOS1*, *IGF1*, *CDKN1A*, *PTEN*, *MAPK1*	0.0114281552734
	miR-379-5p	*MDM2*	
Prostate cancer (hsa05215)	miR-519a-3p	*PDGFRA*, *E2F1*, *E2F2*, *CREB5*, *CDK2*, *CREB1*, *SOS1*, *IGF1*, *PDGFD*, *CDKN1A*, *PTEN*, *MAPK1*	0.0114281552734
	miR-379-5p	*MDM2*	
Oocyte meiosis (hsa04114)	miR-519a-3p	*CDK2*, *PPP3R1*, *CPEB1*, *PPP3CA*, *ITPR1*, *CPEB2*, *RPS6KA3*, *IGF1*, *CPEB3*, *MAPK1*, *FBXW11*	0.0161895429749
	miR-379-5p	*CCNB1*, *PPP2R5D*	
TGF-beta signaling pathway (hsa04350)	miR-519a-3p	*INHBA*, *ZFYVE9*, *SMAD5*, *ACVR1C*, *SMAD7*, *MAPK1*, *TGFBR2*, *BMPR2*	0.0281334933867
	miR-379-5p	*FST*, *TGFBR1*, *LTBP1*	
FoxO signaling pathway (hsa04068)	miR-519a-3p	*STAT3*, *CDK2*, *MAPK8*, *FOXG1*, *SOS1*, *IGF1*, *SOD2*, *CDKN1A*, *PTEN*, *MAPK1*, *CCNG2*, *TGFBR2*	0.0455909431746
	miR-379-5p	*TGFBR1*, *CNB1*, *INSR*, *MDM2*	

**Table 5:** Integrated overview of miRNA, TF, target gene, TF functions, and associated diseases.

miRNAs	Transcription Factor	Target Genes	Function of Transcription Factor	Diseases with Associated Transcription Factor
miR-519a-3p and miR-379-5p	ESR1 (Estrogen receptor 1)	*ABCG2*, *AHR*, *AMH*, *AR*, *AVP*, *BCL2*, *BLM*, *BRCA1*, *BTG2*, *CCNA2*, *CCND1*, *CD24*, *CDH1*, *CDK4*, *CDKN1A*, *CDKN1B*, *CEBPB*, *CHAT*, *CRH*, *CRHBP*, *CTNNB1*, *CTSD*, *CXCL12*, *CYP19A1,CYP1A1*, *CYP1B1*, *CYP2C19,DCT*, *E2F1*, *EGFR*, *ESR1*, *ESRRA*, *F12*, *FLT1*, *FOS*, *FOXP1*, *GREB1*, *HSPB1*, *HTRA2*, *JAK2*, *JUN*, *JUNB*, *KDR*, *KRT19*, *MDM2*, *MICB*, *MMP13*, *MTA3*, *MYC*, *NQO1*, *NR5A2*, *NRF1*, *OXT*, *PELP1*, *PGR*, *PLAC1*, *PMAIP1*, *PTMA*, *RARA*, *RET*, *RUNX2*, *SERPINB9*, *SERPINE1*, *SP1*, *TAC3*, *TERT*, *TFF1*, *TGFA*, *TP53*, *TYMS*, *UGT1A4*, *UGT2B15*, *VEGFA*, *YWHAQ*, *ZEB1*, *ZFHX3*	Regulates of eukaryotic gene expression and affect cellular proliferation and differentiation, association with DNA-binding transcription factors.	Malignant neoplasm of prostate, Malignant neoplasm of ovary, Embryoma, Malignant tumor of colon, Malignant neoplasm of stomach, Primary carcinoma of the liver cells, Breast Carcinoma, Renal Cell Carcinoma, Carcinoma of the Large Intestine, Leukemia, Metastasis to Lymph Nodes
	FOXA1 (Forkhead box A1)	*ABCA1*, *AGR2*, *APOB*, *BCL2*, *BRCA1*, *CCNG2*, *CFTR*, *ESR1*, *GCG*, *HSPA1A*, *KRT7*, *MYC*, *NF1*, *NKX2-1*, *RET*, *RPRM*, *SERPINC1*, *SFTPB*, *TFF1*, *UGT2B15*, *UGT2B17*	Participates in embryogenesis, aids in defining tissue-specific gene expression patterns, and modulates gene regulation in differentiated tissues.	Cancer, Fibroadenoma, Breast Carcinoma, Prostate carcinoma, Myocardial Ischemia, Primary Tumor, Medullary carcinoma of thyroid, Infertility, Congenital Abnormality, Endometrial Carcinoma, Malignant neoplasm of prostate, Primary carcinoma of the liver cells, Malignant neoplasm of male breast, Embryoma, Renal Cell Carcinoma, Neoplasm Metastasis, Breast diseases, Myeloid leukemia, Coronary heart disease, Obesity, Carcinoma of the Large Intestine, Diabetes Mellitus, Laryngeal Squamous Cell Carcinoma, Lung diseases, Bilateral Breast Cancer, Gastrointestinal Stromal Tumors, Malignant neoplasm of pancreas, Malignant tumor of colon, Squamous cell carcinoma
miR-519a-3p	KLF4 (KLF transcription factor 4)	*ALPI*, *ATF3*, *BDKRB2*, *BIRC5*, *CCNB1*, *CCND1*, *CD14*, *CDH1*, *CDH5*, *CDKN1A*, *CDKN1B*, *CDKN1C*, *CDX2*, *CYP1A1*, *GDF15*, *GPA33*, *HBA1*, *HBA2*, *HDC*, *HSPA8*, *IFITM3*, *IL1B*, *IL6*, *IVL*, *KRT19*, *LAMA1*, *LAMA3*, *LXN*, *MMP2*, *NANOG*, *ODC1*, *PFKP*, *RARA*, *SOD1*, *TAGLN*, *THBD*, *TP53*, *VDR*, *VEGFA*, *ZNF750*	Functions as both an activator and a repressor, influencing the expression of critical transcription factors throughout embryonic development.	Carcinoma of the Large Intestine, Thyroid carcinoma, Carcinoma of Nasopharynx, Stomach Carcinoma, Renal Cell Carcinoma, Embryoma, Myeloid leukemia, Breast Carcinoma, Malignant neoplasm of prostate, Leukemia, Malignant neoplasm of lung, Glioma, Malignant neoplasm of ovary, Colorectal Neoplasms, Endometriosis, Cervical Squamous Cell Carcinoma, Malignant tumor of colon, small cell carcinoma of lung, Cervix carcinoma
miR-519a-3p	PGR (Progesterone receptor)	*ABCG2*, *ATP1B1*, *BCL2*, *CCND1*, *CYP19A1*, *DUSP1*, *E2F1*, *EGFR*, *ERBB2*, *ESR1*, *FOXP3*, *HLTF*, *IGFBP1*, *IL10*, *IRS2*, *KLK3*, *KLK4*, *MYC*, *PLD1*, *PTGS2*, *RELA*, *RLN1*, *RLN2*, *VEGFA*, *YWHAQ*	Regulates of eukaryotic gene expression and affect cellular proliferation and differentiation in target tissues.	Malignant neoplasm of ovary, Breast Carcinoma, Embryoma, Malignant tumor of colon, Uterine Fibroids, Melanoma, Primary carcinoma of the liver cells, Renal Cell Carcinoma, Squamous Cell Carcinoma of Esophagus, Stomach Carcinoma, Endometrial Carcinoma, Neuroblastoma, Carcinoma of the Large Intestine, Endometriosis, Infiltrating Malignant Neoplasm, Prostate carcinoma Parkinson disease
miR-379-5p	STAT1 (Signal transducer and activator of transcription 1)	*ACAT1*, *APP*, *BAX*, *CCL2*, *CCL3*, *CCR1*, *CD22*, *CD40*, *CD40LG*, *CD86*, *CDKN1A*, *CEBPE*, *CFTR*, *CHAT*, *CIITA*, *CTSB*, *CTSL*, *CXCL10*, *EDN1*, *EGFR*, *FAS*, *FCGR1A*, *FCGRT*, *FGF2*, *FGFR3*, *FOXP3*, *GAST*, *GLS*, *HLA-E*, *HMOX1*, *HSP90AA1*, *ICAM1*, *IFIT3*, *IFNA1*, *IFNA1*, *IFNB1*, *IFNG*, *IFNLR1*, *IL10*, *IL1B*, *IL1R1*, *IL27*, *IL2RA*, *IL6*, *IRF1*, *IRF7*, *IRF8*, *IVL*, *JAK2*, *KRT17*, *LCN2*, *LY96*, *MMP13*, *MMP9*, *MUC1*, *MUC4*, *MVP*, *NOS2*, *NOX1*, *NOX4*, *NOX5*, *NR1H4*, *OPRM1*, *PIM1*, *PLSCR1*, *PML*, *PPARA*, *PRKCE*, *PSMB9*, *PTGS2*, *PTGS2*, *S100A10*, *SCARB1*, *SMARCA4*, *SOCS3*, *STAT2*, *STAT3*, *TAP1*, *TBX21*, *TIMP1*, *TLR3*, *TNFSF13B*, *TP53*, *UPP1*, *VIP*, *XAF1*	Mediates cellular responses by interferons (IFNs), the cytokine KITLG/SCF, and other cytokines and other growth factors.	Rheumatoid Arthritis, Malignant neoplasm of breast, Squamous cell carcinoma, Malignant neoplasm of prostate, Asthma, Melanoma, Myeloid leukemia, Systemic lupus erythematosus, Lymphoma, Leukemia, Carcinoma of the Large Intestine, Endometriosis, Diabetes Mellitus, Primary carcinoma of the liver cells, Malignant tumor of colon, Glioblastoma, Multiple Myeloma, Stomach Carcinoma
miR-379-5p	SMAD2 (SMAD family member 2)	*SMAD2*, *BCL2*, *CDKN1A*, *GLI1*, *NKX2-1*	Modulator activator of intracellular signal transducer	Gastrointestinal Stromal Tumors, Carcinoma of lung, Malignant neoplasm of stomach, Malignant neoplasm of esophagus, Metastasis to Lymph Nodes, Lymphoma, Leiomyosarcoma, Malignant Glioma, Neuroendocrine Tumors, Sarcoma, Laryngeal Squamous Cell Carcinoma, Malignant tumor of colon, Fibroid Tumor, Melanoma, Cervix carcinoma, Thyroid carcinoma, Myeloid leukemia
miR-379-5p	HNF4A (Hepatocyte nuclear factor 4 alpha)	*ABCC6*, *ABCG5*, *ABCG8*, *ACAT2*, *AFP*, *AKR1C4*, *APOA1*, *APOA2*, *APOB*, *APOC3*, *C1QTNF5*, *CDKN1A*, *CEACAM1*, *CYP27A1*, *CYP2B6*, *CYP2C8*, *CYP2C9*, *CYP2D6*, *CYP3A4*, *CYP7A1*, *FABP2*, *GCK*, *GFER*, *GH1*, *GPR39*, *HNF1A*, *CNJ11*, *LDLR*, *LIPC*, *MP7*, *MTTP*, *MYC*, *PC1L1*, *NR1I2*, *PCSK9*, *PPARA*, *RNASE2*, *SERPINC1*, *SHBG*, *SLC22A6*, *SLC26A3*, *TBP*, *TCF7L2*, *UGT1A7*, *UGT2B7*	Transcriptional regulation/Regulates the recruitment of RNA pol II to the promoters of target genes	Hypercholesterolemia, Coronary Arteriosclerosis, Liver neoplasms, Obesity, Diabetes Mellitus, Endometrial Carcinoma, Bile duct carcinoma, Epithelial ovarian cancer, Myoma, Breast Carcinoma, Polycystic Ovary syndrome, Renal Cell Carcinoma, Skin Melanoma, Malignant neoplasm of prostate, Ulcerative colitis, Neuroendocrine Tumors, Malignant tumor of colon, Laryngeal Squamous Cell Carcinoma
miR-379-5p	MAZ (MYC associated zinc finger protein)	*CD4*, *CLCNKA*, *HRAS*, *HTR1A*, *MMP1*, *MMP14*, *MMP9*, *TSG101*	Transcriptional regulator	Epithelial ovarian cancer, Embryoma, Malignant neoplasm of breast, Cervical Squamous Cell Carcinoma, Carcinoma of the Large Intestine, Diabetes Mellitus, Metastasis to Lymph Nodes, Carcinoma of larynx, Endometrial Carcinoma, Hereditary Nonpolyposis Colorectal Neoplasms, Renal Cell Carcinoma, Colorectal Neoplasms
miR-379-5p	JUN (Jun proto-oncogene, AP-1 transcription factor subunit)	*ABCB1*, *ALOX12*, *APOC3*, *APP*, *AR*, *ATF3*, *BATF3*, *BCL2L1*, *BECN1*, *BEX2*, *BRCA1*, *CA2*, *CCK*, *CCL2*, *CCL5*, *CCND1*, *CD82*, *CDK5R1*, *CDKN1A*, *CREM*, *CSF1*, *CSF2*, *CSTA*, *CTGF*, *CTSL*, *CXCL8*, *CYP11A1*, *CYP19A1*, *CYP1A2*, *CYP2J2*, *DCN*, *DDIT3*, *DDX21*, *EDN1*, *EGFR*, *ELN*, *ETS1*, *ETS2*, *EZH2*, *EZR*, *F3*, *FAS*, *FASLG*, *FGF7*, *VEGFD*, *FOSL1*, *GCLC*, *GJA1*, *GSTP1*, *HEY1*, *IBSP*, *IFNB1*, *IFNG*, *IL12A*, *IL12B*, *IL1A*, *IL1B*, *IL2*, *IL23A*, *IL24*, *IL3*, *IL5RA*, *IL6*, *ITGAX*, *ITGB8*, *JUN*, *KRT16*, *LBP*, *LDHA*, *LGALS3*, *LOR*, *MAP3K1*, *MAPK8*, *MAT2A*, *MEF2D*, *MELTF*, *MGMT*, *MGP*, *MMP1*, *MMP12*, *MMP13*, *MMP2*, *MMP20*, *MMP3*, *MMP7*, *MMP9*, *MSR1*, *MSR1*, *MYB*, *MYC*, *NAMPT*, *NEFL*, *NEIL1*, *NFKB2*, *NGF*, *NOS2*, *NOS3*, *NOX5*, *NPY*, *NQO1*, *NTS*, *OPRM1*, *OXTR*, *PCK2*, *PDHA1*, *PDK1*, *PGR*, *PPARA*, *PTN*, *RHOB*, *RUNX2*, *SLC19A1*, *SMAD7*, *SOD2*, *SOX7*, *SPI1*, *STAR*, *TERT*, *TNF*, *TP53*, *TXN*, *VDR*, *VEGFA*	Activator/Transcription regulation	Malignant neoplasm of prostate, Melanoma, Malignant neoplasm of breast, Neoplasm Metastasis, Embryoma, Carcinoma of the Large Intestine, Malignant neoplasm of stomach, Squamous cell carcinoma, Malignant neoplasm of pancreas, Rheumatoid Arthritis, Malignant neoplasm of urinary bladder, Endometrial Carcinoma, Malignant tumor of colon, Myeloid leukemia, Multiple Sclerosis
miR-379-5p	ATF1 (Activating transcription factor 1)	*BCL2*, *CCNA1*, *CFTR*, *CGA*, *CYP11B1*, *CYP11B2*, *ERBB2*, *FLT1*, *FTH1*, *GABPA*, *IL10*, *LDHA*, *MAP1LC3B*, *MITF*, *MUC2*, *NTS*, *PCSK1*, *PLAUR*, *SLC20A1*, *SLC22A8*, *TGFB2*, *TH*, *TOP2A*, *USP7*	Regulates cell proliferation and transformation	Malignant tumor of colon, Stomach Neoplasms, Carcinoma of lung, Melanoma, Cholangiocarcinoma, Peptic Ulcer, Malignant neoplasm of prostate, Carcinoma of the Large Intestine, Adenocarcinoma of the Pancreas, Fibroadenoma, Malignant Squamous Cell Neoplasm, Small cell carcinoma of lung, Leukemia, Malignant neoplasm of cervix uteri, Glioblastoma, Colorectal Neoplasms
miR-379-5p	EP300 (E1A binding protein p300)	*ABCB1*, *ALOX15*, *BIRC5*, *BRCA1*, *CA9*, *CCNB2*, *CCND1*, *CCNE1*, *CDKN1A*, *CDKN2B*, *COL1A2*, *CRABP1*, *CREBBP*, *CXCL8*, *CYBB*, *CYP1B1*, *DNMT1*, *EIF2AK1*, *EPO*, *ERBB2*, *EZH2*, *GFAP*, *HINFP*, *IFNG*, *IGF1*, *IGFBP3*, *IL12B*, *IL5*, *IL6*, *KLK3*, *KRT16*, *LAMA3*, *MDM2*, *MGMT*, *MMP9*, *MYC*, *NFKB1*, *NR0B2*, *NR1H4*, *PARP1*, *PCNA*, *PTGS2*, *RAD51*, *RELA*, *RS1*, *S100A4*, *SLC9A2*, *TBXAS1*, *THBD*, *TP73*, *TRIM22*, *VCAM1*, *VEGFA*, *WT1*, *YY1*, *ZEB1*	Regulates transcription via chromatin remodeling	Malignant neoplasm of breast, Embryoma, Malignant Neoplasms, Carcinoma of the Large Intestine, Squamous cell carcinoma, Malignant neoplasm of lung, Endometrial Carcinoma, Melanoma, Colorectal Neoplasms, Malignant neoplasm of ovary, Malignant neoplasm of prostate, Glioblastoma, Leukemia, Malignant tumor of colon, Meningioma

## Data Availability

The datasets used and/or analyzed during the current study are available from the corresponding author on reasonable request.

## Supplementary Materials

The following supporting information can be downloaded at: https://www.mdpi.com/article/10.3390/diagnostics15162023/s1, Table S1: GO enrichment annotations of miR-379-5p and miR-519a-3p (A): Biological Process (BP), (B): Cellular Component (CC), (C): Molecular Function (MF).

## Abbreviations

The following abbreviations are used in this manuscript:

AKT3	AKT serine/threonine kinase 3
APC	Adenomatous polyposis coli
ATF1	Activating transcription factor 1
CASP3	Caspase 3
CRC	Colorectal cancer
ceRNAs	Competitive endogenous RNAs
EMT	Epithelial–mesenchymal transition
ECM	Extracellular matrix
EP300	E1A binding protein p300
ESR1	Estrogen receptor 1
FOXA1	Forkhead box A1
GO	Gene Ontology
HNF4A	Hepatocyte nuclear factor 4 alpha
JUN	Jun proto-oncogene, AP-1 transcription factor subunit
KEGG	Kyoto Encyclopedia of Genes and Genomes
KLF4	KLF transcription factor 4
MAZ	MYC associated zinc finger protein
miRNA	microRNA
NK	Natural killer
PGR	Progesterone receptor
RT-qPCR	Quantitative Real-time PCR
SMAD2	SMAD family member 2
STAT1	Signal transducer and activator of transcription 1
STAT3	Signal transducer and activator of transcription 3
ROR1	Receptor Tyrosine Kinase Like Orphan Receptor 1
TF	Transcription factors
TGF-β	Transforming growth factor beta

## References

[1] Siegel R.L., Giaquinto A.N., Jemal A. (2024). Cancer statistics, 2024. CA A Cancer J. Clin..

[2] Ozaslan M., Aytekin T. (2009). Loss of heterozygosity in colorectal cancer. Afr. J. Biotechnol..

[3] Sawicki T., Ruszkowska M., Danielewicz A., Niedźwiedzka E., Arłukowicz T., Przybyłowicz K.E. (2021). A Review of Colorectal Cancer in Terms of Epidemiology, Risk Factors, Development, Symptoms and Diagnosis. Cancers.

[4] Kim T., Croce C.M. (2023). MicroRNA: Trends in clinical trials of cancer diagnosis and therapy strategies. Exp. Mol. Med..

[5] Avsar R., Gurer T., Aytekin A. (2023). Bioinformatics and Expression Analyses of miR-639, miR-641, miR-1915-3p and miR-3613-3p in Colorectal Cancer Pathogenesis. J. Cancer.

[6] Zhang Z., Liu X., Feng B., Liu N., Wu Q., Han Y., Nie Y., Wu K., Shi Y., Fan D. (2015). STIM1, a direct target of microRNA-185, promotes tumor metastasis and is associated with poor prognosis in colorectal cancer. Oncogene.

[7] Forterre A., Komuro H., Aminova S., Harada M. (2020). A Comprehensive Review of Cancer MicroRNA Therapeutic Delivery Strategies. Cancers.

[8] Yang B., Xia S., Ye X., Jing W., Wu B. (2021). MiR-379-5p targets microsomal glutathione transferase 1 (MGST1) to regulate human glioma in cell proliferation, migration and invasion and epithelial-mesenchymal transition (EMT). Biochem. Biophys. Res. Commun..

[9] Aytekin A., Kadakal H., Mihcioglu D., Gurer T. (2025). Bioinformatics analysis of miR-2861 and miR-5011-5p that function as potential tumor suppressors in colorectal carcinogenesis. BMC Med. Genom..

[10] Mihcioglu D., Elihan E., Aytekin A., Gurer T. (2023). miR-564 and miR-718 expressions are downregulated in colorectal cancer tissues. Turk. J. Biochem..

[11] Yu W., Wang J., Li C., Xuan M., Han S., Zhang Y., Liu P., Zhao Z. (2022). miR-17-5p promotes the invasion and migration of colorectal cancer by regulating HSPB2. J. Cancer.

[12] Liang M., Chen H., Min J. (2021). miR-379-5p inhibits proliferation and invasion of the endometrial cancer cells by inhibiting expression of ROR1. Acta Biochim. Pol..

[13] Shukla D., Mishra S., Mandal T., Charan M., Verma A.K., Khan M.M.A., Chatterjee N., Dixit A.K., Ganesan S.K., Ganju R.K. (2025). MicroRNA-379-5p attenuates cancer stem cells and reduces cisplatin resistance in ovarian cancer by regulating RAD18/Polη axis. Cell Death Dis..

[14] Meng L., Du Y., Deng B., Duan Y. (2023). miR-379-5p regulates the proliferation, cell cycle, and cisplatin resistance of oral squamous cell carcinoma cells by targeting ROR1. Am. J. Transl. Res..

[15] Wu D., Niu X., Tao J., Li P., Lu Q., Xu A., Chen W., Wang Z. (2017). MicroRNA-379-5p plays a tumor-suppressive role in human bladder cancer growth and metastasis by directly targeting MDM2. Oncol Rep..

[16] Chen J.S., Li H.S., Huang J.Q., Dong S.H., Huang Z.J., Yi W., Zhan G.F., Feng J.T., Sun J.C., Huang X.H. (2016). MicroRNA-379-5p inhibits tumor invasion and metastasis by targeting FAK/AKT signaling in hepatocellular carcinoma. Cancer Lett..

[17] Qiu S., Xie L., Lu C., Gu C., Xia Y., Lv J., Xuan Z., Fang L., Yang J., Zhang L. (2022). Gastric cancer-derived exosomal miR-519a-3p promotes liver metastasis by inducing intrahepatic M2-like macrophage-mediated angiogenesis. J. Exp. Clin. Cancer Res..

[18] Breunig C., Pahl J., Küblbeck M., Miller M., Antonelli D., Erdem N., Wirth C., Will R., Bott A., Cerwenka A. (2017). MicroRNA-519a-3p mediates apoptosis resistance in breast cancer cells and their escape from recognition by natural killer cells. Cell Death Dis..

[19] Ren L., Li Y., Zhao Q., Fan L., Tan B., Zang A., Yang H. (2020). miR-519 regulates the proliferation of breast cancer cells via targeting human antigen R. Oncol. Lett..

[20] Wang G., Yu Y., Wang Y. (2022). Effects of propofol on neuroblastoma cells via the HOTAIRM1/miR-519a-3p axis. Transl. Neurosci..

[21] Jácome D., Cotrufo T., Andrés-Benito P., Lidón L., Martí E., Ferrer I., Del Río J.A., Gavín R. (2024). miR-519a-3p, found to regulate cellular prion protein during Alzheimer’s disease pathogenesis, as a biomarker of asymptomatic stages. Biochim. Biophys. Acta Mol. Basis Dis..

[22] Tolosa E., Botta-Orfila T., Morató X., Calatayud C., Ferrer-Lorente R., Martí M.-J., Fernández M., Gaig C., Raya Á., Consiglio A. (2018). MicroRNA alterations in iPSC-derived dopaminergic neurons from Parkinson disease patients. Neurobiol. Aging.

[23] Gurer T., Aytekin A., Caki E., Gezici S. (2022). miR-485-3p and miR-4728-5p as Tumor Suppressors in Pathogenesis of Colorectal Cancer. Mol. Biol..

[24] Livak K.J., Schmittgen T.D. (2001). Analysis of relative gene expression data using real-time quantitative PCR and the 2(-Delta Delta C(T)) Method. Methods.

[25] Chen W., Gao C., Liu Y., Wen Y., Hong X., Huang Z. (2020). Bioinformatics Analysis of Prognostic miRNA Signature and Potential Critical Genes in Colon Cancer. Front. Genet..

[26] Kanehisa M., Furumichi M., Tanabe M., Sato Y., Morishima K. (2017). KEGG: New perspectives on genomes, pathways, diseases and drugs. Nucleic Acids Res..

[27] Liang M., Zhang C., Yang Y., Cui Q., Zhang J., Cui C. (2025). TransmiR v3.0: An updated transcription factor-microRNA regulation database. Nucleic Acids Res..

[28] Han H., Cho J.W., Lee S., Yun A., Kim H., Bae D., Yang S., Kim C.Y., Lee M., Kim E. (2018). TRRUST v2: An expanded reference database of human and mouse transcriptional regulatory interactions. Nucleic Acids Res..

[29] The Human Protein Atlas. https://www.proteinatlas.org.

[30] Shen Y.T., Huang X., Zhang G., Jiang B., Li C.J., Wu Z.S. (2021). Pan-Cancer Prognostic Role and Targeting Potential of the Estrogen-Progesterone Axis. Front. Oncol..

[31] Liu N., Wang A., Xue M., Zhu X., Liu Y., Chen M. (2024). FOXA1 and FOXA2: The regulatory mechanisms and therapeutic implications in cancer. Cell Death Discov..

[32] He Z., He J., Xie K. (2023). KLF4 transcription factor in tumorigenesis. Cell Death Discov..

[33] Li M., Zhou C. (2021). Progesterone receptor gene serves as a prognostic biomarker associated with immune infiltration in gastric cancer: A bioinformatics analysis. Transl. Cancer Res..

[34] Meissl K., Macho-Maschler S., Müller M., Strobl B. (2017). The good and the bad faces of STAT1 in solid tumours. Cytokine.

[35] Wang Q., Xiong F., Wu G., Wang D., Liu W., Chen J., Qi Y., Wang B., Chen Y. (2023). SMAD Proteins in TGF-β Signalling Pathway in Cancer: Regulatory Mechanisms and Clinical Applications. Diagnostics.

[36] Qu N., Luan T., Liu N., Kong C., Xu L., Yu H., Kang Y., Han Y. (2023). Hepatocyte nuclear factor 4 a (HNF4α): A perspective in cancer. Biomed. Pharmacother..

[37] Maity G., Haque I., Ghosh A., Dhar G., Gupta V., Sarkar S., Azeem I., McGregor D., Choudhary A., Campbell D.R. (2018). The MAZ transcription factor is a downstream target of the oncoprotein Cyr61/CCN1 and promotes pancreatic cancer cell invasion via CRAF-ERK signaling. J. Biol. Chem..

[38] Jafri Z., Li Y., Zhang J., O’Meara C.H., Khachigian L.M. (2025). Jun, an Oncological Foe or Friend?. Int. J. Mol. Sci..

[39] Lu Z., Dong H., Tu Z., Liu H. (2025). Expression, molecular mechanisms and therapeutic potentials of ATF1 in cancers. Life Sci..

[40] Gronkowska K., Robaszkiewicz A. (2024). Genetic dysregulation of EP300 in cancers in light of cancer epigenome control—Targeting of p300-proficient and-deficient cancers. Mol. Ther. Oncol..

[41] Gururajan M., Josson S., Chu G.C., Lu C.L., Lu Y.T., Haga C.L., Zhau H.E., Liu C., Lichterman J., Duan P. (2014). miR-154 and miR-379 in the DLK1-DIO3 microRNA mega-cluster regulate epithelial to mesenchymal transition and bone metastasis of prostate cancer. Clin. Cancer Res..

[42] Mosaad H., Abdelrahman A.E., Abdullatif A., Lashin M.E., Ramadan M.S.H., El-Azony A., Hussien M.H.S. (2023). MIR-379-5p Expression in Endometrial Cancer and Its Correlation with ROR1 Expression. Asian Pac. J. Cancer Prev..

[43] Zhang H., Zheng W., Li D., Zheng J. (2022). MiR-379-5p Promotes Chondrocyte Proliferation via Inhibition of PI3K/Akt Pathway by Targeting YBX1 in Osteoarthritis. Cartilage.

[44] Maemura K., Watanabe K., Ando T., Hiyama N., Sakatani T., Amano Y., Kage H., Nakajima J., Yatomi Y., Nagase T. (2018). Altered editing level of microRNAs is a potential biomarker in lung adenocarcinoma. Cancer Sci..

[45] Lv X., Wang M., Qiang J., Guo S. (2019). Circular RNA circ-PITX1 promotes the progression of glioblastoma by acting as a competing endogenous RNA to regulate miR-379-5p/MAP3K2 axis. Eur. J. Pharmacol..

[46] Gu Z., Wu S., Wang J., Zhao S. (2020). Long non-coding RNA LINC01419 mediates miR-519a-3p/PDRG1 axis to promote cell progression in osteosarcoma. Cancer Cell Int..

[47] Li H., Chen L., Li J.-J., Zhou Q., Huang A., Liu W.-W., Wang K., Gao L., Qi S.-T., Lu Y.-T. (2018). miR-519a enhances chemosensitivity and promotes autophagy in glioblastoma by targeting STAT3/Bcl2 signaling pathway. J. Hematol. Oncol..

[48] Wang Y., Jiang F., Wang J., Fu Y., Li Y., Li F. (2020). MiR-519a functions as a tumor suppressor and is negatively associated with poor prognosis of non-small cell lung cancer. Cancer Biomark..

[49] Nong K., Zhang D., Chen C., Yang Y., Yang Y., Liu S., Cai H. (2020). MicroRNA-519 inhibits hypoxia-induced tumorigenesis of pancreatic cancer by regulating immune checkpoint PD-L1. Oncol Lett..

[50] Ward A., Shukla K., Balwierz A., Soons Z., König R., Sahin O., Wiemann S. (2014). MicroRNA-519a is a novel oncomir conferring tamoxifen resistance by targeting a network of tumour-suppressor genes in ER+ breast cancer. J. Pathol..

[51] Liebl M.C., Hofmann T.G. (2021). The Role of p53 Signaling in Colorectal Cancer. Cancers.

[52] Fasano M., Pirozzi M., Miceli C.C., Cocule M., Caraglia M., Boccellino M., Vitale P., De Falco V., Farese S., Zotta A. (2024). TGF-β Modulated Pathways in Colorectal Cancer: New Potential Therapeutic Opportunities. Int. J. Mol. Sci..

[53] Laissue P. (2019). The forkhead-box family of transcription factors: Key molecular players in colorectal cancer pathogenesis. Mol. Cancer.

[54] Zhou M., Liu X., Li Z., Huang Q., Li F., Li C.Y. (2018). Caspase-3 regulates the migration, invasion and metastasis of colon cancer cells. Int. J. Cancer.

[55] Grant A., Xicola R.M., Nguyen V., Lim J., Thorne C., Salhia B., Llor X., Ellis N., Padi M. (2021). Molecular drivers of tumor progression in microsatellite stable APC mutation-negative colorectal cancers. Sci. Rep..

[56] Fang Y., Liang X., Xu J., Cai X. (2018). miR-424 targets AKT3 and PSAT1 and has a tumor-suppressive role in human colorectal cancer. Cancer Manag. Res..

[57] Hao S., Huo S., Du Z., Yang Q., Ren M., Liu S., Liu T., Zhang G. (2019). MicroRNA-related transcription factor regulatory networks in human colorectal cancer. Medicine.

[58] Qin G., Mallik S., Mitra R., Li A., Jia P., Eischen C.M., Zhao Z. (2020). MicroRNA and transcription factor co-regulatory networks and subtype classification of seminoma and non-seminoma in testicular germ cell tumors. Sci. Rep..

[59] Rawłuszko-Wieczorek A.A., Marczak Ł., Horst N., Horbacka K., Krokowicz P., Jagodziński P.P. (2017). Significance of intratissue estrogen concentration coupled with estrogen receptors levels in colorectal cancer prognosis. Oncotarget.

[60] Lazar S.B., Pongor L., Li X.L., Grammatikakis I., Muys B.R., Dangelmaier E.A., Redon C.E., Jang S.M., Walker R.L., Tang W. (2020). Genome-Wide Analysis of the FOXA1 Transcriptional Network Identifies Novel Protein-Coding and Long Noncoding RNA Targets in Colorectal Cancer Cells. Mol. Cell. Biol..

[61] Hussen B.M., Hidayat H.J., Salihi A., Sabir D.K., Taheri M., Ghafouri-Fard S. (2021). MicroRNA: A signature for cancer progression. Biomed. Pharmacother..

[62] Yu W., Wang Z., Shen L.I., Wei Q. (2016). Circulating microRNA-21 as a potential diagnostic marker for colorectal cancer: A meta-analysis. Mol. Clin. Oncol..

[63] Toiyama Y., Hur K., Tanaka K., Inoue Y., Kusunoki M., Boland C.R., Goel A. (2014). Serum miR-200c is a novel prognostic and metastasis-predictive biomarker in patients with colorectal cancer. Ann. Surg..

[64] Sur D., Advani S., Braithwaite D. (2022). MicroRNA panels as diagnostic biomarkers for colorectal cancer: A systematic review and meta-analysis. Front. Med..

