# Supplementary material for: Expression Analysis of miR-519a-3p and miR-379-5p in Colorectal Cancer Patients: A Combined Experimental and Bioinformatic Approach
Source: Diagnostics (Basel). 2025 Aug 13;15(16):2023. doi: 10.3390/diagnostics15162023 (PMC12386024; doi:10.3390/diagnostics15162023)
Supplement: Supplementary file 1 [file diagnostics-15-02023-s001.zip › diagnostics-3745259-supplementary.pdf]

SUPPLEMENTARY MATERIAL

Supplementary Table S1. GO enrichment annotations of miR-379-5p and miR-519a-3p (A): Biological Process (BP), (B): Cellular Component (CC), (C): Molecular Function (MF).

A

GO Category	miRNAs	Target Genes
Cellular nitrogen compound metabolic process (GO:0034641)	miR-519a-3p	*MYBL1, ZNF19, SUGP2, DYNC1H1, SFMBT1, MED17, KLF10, RASD1, ZXDB, ARL1, HIRA, HSPA8, ZNF514, CBX7, ZBTB4, ARX, KDM5B*
	miR-379-5p	*POU2F1, KIF20A, PRPF40A, ANPEP, HUWE1, MNT, TUBB, AGO2, HMOX2, COPS2, TAOK1, MME, NFAT5, POLR2A, CASP3, DM4A, IGFBP4, ADM, MGMT, IGF2, RPS16, EIF4G1, PABPC1HSPA8, BCL6, NFE2L1, DDX39B, ZFHX4, CAD, CREBBP, SERPINE1, BAZ2B, LMO4, SIN3A*
Cellular protein metabolic process (GO:0044267)	miR-519a-3p	*TLN1*
	miR-379-5p	*ANPEP, PAPPA, GALNT11, PDIA6, MME, IGFBP4, IGF2, RPS16, EIF4G1, PABPC1*
Biosynthetic process (GO:0009058)	miR-519a-3p	*MYBL1, ZNF19, FMBT1, MED17, KLF10, ZXDB, HIRA, HSPA8, ZNF514, CBX7, ZBTB4, ARX, PTEN, KDM5B*
	miR-379-5p	*POU2F1, MGST1, MNT, AGO2, COPS2, NFAT5, POLR2A, KDM4A, GCN1L1, ADM, COL5A1, RPS16, EIF4G1, PABPC1, LUM, HSPA8, BCL6, NFE2L1, GPI, ZFHX4, CAD, CREBBP, SERPINE1, BAZ2B, LMO4, SIN3A*
Response to stress (GO:0006950)	miR-519a-3p	*DNAJA1, APC, KLF10, TNRC6B, KDM6B, HSPA8, CDKN1A, ZBTB4, PTEN, TLN1*
	miR-379-5p	*MGST1, CCNA2, MCL1, MAPKAP1, HUWE1, TUBB, AGO2, HMOX2, PDIA6, TAOK1, UBE2L6, RIF1, POLR2A, MGMT, HSPA8, BCL6, NFE2L1, CREBBP, SERPINE1*
Symbiosis, encompassing mutualism through parasitism (GO:0044403)	miR-519a-3p	*UBE3A, HSPA8, AP2S1*
	miR-379-5p	*ANPEP, ITGA5, POLR2A, KDM4A, RPS16, EIF4G1, HSPA8, DDX39B, CREBBP*
Viral process (GO:0016032)	miR-519a-3p	*UBE3A, HSPA8, AP2S1*
	miR-379-5p	*ANPEP, ITGA5, POLR2A, KDM4A, RPS16, EIF4G1, HSPA8, CREBBP*
Gene expression (GO:0010467)	miR-519a-3p	*MED17, TNRC6B, HSPA8*
	miR-379-5p	*POU2F1, AGO2, POLR2A, RPS16, EIF4G1, PABPC1, HSPA8, CREBBP, SERPINE1*
Macromolecular complex assembly (GO:0065003)	miR-519a-3p	*APC, DYNC1H1, HIRA, FARP2, AP2S1*
	miR-379-5p	*MGST1, COA1, TUBB, COL6A1, FIS1, SHKBP1, DDX39B, CREBBP, HIST1H3B, HIST1H3C*
Neurotrophin TRK receptor signaling pathway (GO:0048011)	miR-519a-3p	*TNRC6B, CDKN1A, PTEN, AP2S1*
	miR-379-5p	*MAPKAP1, AGO2, CASP3*
Protein complex assembly (GO:0006461)	miR-519a-3p	*APC, HIRA, FARP2, AP2S1*
	miR-379-5p	*MGST1, COA1, TUBB, COL6A1, FIS1, SHKBP1, CREBBP, HIST1H3B, HIST1H3C*
Cellular component assembly (GO:0022607)	miR-519a-3p	*APC, DYNC1H1, HIRA, FARP2, PTEN, AP2S1, TLN1*
	miR-379-5p	*MGST1, COA1, TUBB, COL6A1, FIS1, SHKBP1, DDX39B, CREBBP, HIST1H3B, HIST1H3C*

Fc-epsilon receptor signaling pathway (GO:0038095)	miR-519a-3p	*TNRC6B, CDKN1A, PTEN*
	miR-379-5p	*MAPKAP1, AGO2*
Catabolic process (GO:0009056)	miR-519a-3p	*DYNC1H1, CACUL1, RASD1, ARL1, UBE3A, HSPA8, PTEN*
	miR-379-5p	*KIF20A, TUBB, HMOX2, COL6A1, FIS1, CASP3, COL5A1, RPS16, EIF4G1, PABPC1, LUM, HSPA8, DDX39B, GPI, CAD*
Fibroblast growth factor receptor signaling pathway (GO:0008543)	miR-519a-3p	*TNRC6B, SHOC2, CDKN1A, PTEN*
	miR-379-5p	*MAPKAP1, AGO2*
Positive regulation of nuclear-transcribed mRNA catabolic process, deadenylation-dependent decay (GO:1900153)	miR-519a-3p	*TNRC6B*
	miR-379-5p	*AGO2, PABPC1*
Epidermal growth factor receptor signaling pathway (GO:0007173)	miR-519a-3p	*TNRC6B, CDKN1A, PTEN, AP2S1*
	miR-379-5p	*MAPKAP1, AGO2*
Phosphatidylinositol-mediated signaling (GO:0048015)	miR-519a-3p	*TNRC6B, CDKN1A, PTEN*
	miR-379-5p	*MAPKAP1, AGO2*
Positive regulation of nuclear-transcribed mRNA poly(A) tail shortening (GO:0060213)	miR-519a-3p	*TNRC6B*
	miR-379-5p	*AGO2, PABPC1*
Blood coagulation (GO:0007596)	miR-519a-3p	*TLN1*
	miR-379-5p	*ITGA5, IGF2, TFPI2, SERPINE1, SIN3A, HIST1H3B, HIST1H3C*
Aging (GO:0007568)	miR-519a-3p	*TIMP2, CDKN1A, PTEN*
	miR-379-5p	*MME, ADM, SERPINE1, SIN3A*
Cellular protein modification process (GO:0006464)	miR-519a-3p	*PTPN4, FEM1C, UBE3A, CSNK1A1, KDM6B, AKT3, CDKN1A, PTEN, KDM5B*
	miR-379-5p	*HUWE1, WNK1, GALNT11, COPS2, TAOK1, UBE2L6, KDM4A, CAD, CREBBP, HECTD4, GAK, TMEM165, SIN3A*
Clathrin coat disassembly (GO:0072318)	miR-519a-3p	*HSPA8*
	miR-379-5p	*HSPA8, GAK*
Vesicle-mediated transport (GO:0016192)	miR-519a-3p	*ARL1, HSPA8, ARCN1, FAM109B, SNX5, AP2S1, TLN1*
	miR-379-5p	*KIF20A, PDIA6, ADM, IGF2, HSPA8, COPA, SERPINE1, AP3M1*
Histone demethylation (GO:0016577)	miR-519a-3p	*KDM6B*
	miR-379-5p	*KDM4A*
Extracellular matrix disassembly (GO:0022617)	miR-519a-3p	*TIMP2*
	miR-379-5p	*COL6A1, CASP3, COL5A1*
COPI coating of Golgi vesicle (GO:0048205)	miR-519a-3p	*ARCN1*
	miR-379-5p	*COPA*
	miR-519a-3p	*CDKN1A*

Organ regeneration (GO:0031100)	miR-379-5p	*CCNA2, ADM, CAD*
Angiotensin maturation (GO:0002003)	miR-379-5p	*ANPEP, MM*
Negative regulation of cyclin-dependent protein serine/threonine kinase activity (GO:0045736)	miR-519a-3p	*APC, CDKN1A*
	miR-379-5p	*CASP3*
Cellular response to DNA damage stimulus (GO:0006974)	miR-519a-3p	*APC, CDKN1A, ZBTB4*
	miR-379-5p	*TAOK1, RIF1, CASP3, MGMT, BCL6*
Extracellular matrix organization (GO:0030198)	miR-519a-3p	*TIMP2*
	miR-379-5p	*ITGA5, COL6A1, CASP3, COL5A1, LUM, SERPINE1*
Nucleobase-containing compound catabolic process (GO:0034655)	miR-519a-3p	*DYNC1H1, RASD1, ARL1, HSPA8*
	miR-379-5p	*KIF20A, TUBB, CASP3, RPS16, EIF4G1, PABPC1, HSPA8, DDX39B*
Cell-substrate junction assembly (GO:0007044)	miR-519a-3p	*TLN1*
	miR-379-5p	*ITGA5*
Axon guidance (GO:0007411)	miR-519a-3p	*FARP2, HSPA8, ARX, AP2S1, TLN1*
	miR-379-5p	*ITGA5, COL6A1, COL5A1, HSPA8*
Platelet degranulation (GO:0002576)	miR-519a-3p	*TLN1*
	miR-379-5p	*IGF2, SERPINE1*
Regulation of cell cycle (GO:0051726)	miR-519a-3p	*APC, HSPA8, CDKN1A, PTEN*
	miR-379-5p	*CCNA2, MNT, HSPA8*
Androgen receptor signaling pathway (GO:0030521)	miR-519a-3p	*DNAJA1, MED17, UBE3A*
Regulation of gene silencing (GO:0060968)	miR-379-5p	*HIST1H3B, HIST1H3C*
Female pregnancy (GO:0007565)	miR-379-5p	*PAPPA, ADM, IGF2, CAD*
Mitotic cell cycle (GO:0000278)	miR-519a-3p	*DYNC1H1, CDKN1A*
	miR-379-5p	*CCNA2, KIF20A, TUBB, TAOK1*
Response to organonitrogen compound (GO:0010243)	miR-519a-3p	*CDKN1A*
	miR-379-5p	*MGST1, SIN3A*
G2/M transition of mitotic cell cycle (GO:0000086)	miR-519a-3p	*DYNC1H1, CDKN1A*
	miR-379-5p	*CCNA2, TUBB*
Prostate gland growth (GO:0060736)	miR-519a-3p	*UBE3A, PTEN*
**Biological process (GO:0008150): 114 Target Genes**		

B

GO Category	miRNAs	Target Genes
Organelle (GO:0043226)	miR-519a-3p	*MYBL1, DNAJA1, ZNF19, APC, SUGP2, PTPN4, DYNC1H1, SFMBT1, TIMP2, MED17, KLF10, ZXDB, ARL1, PFN2, UBE3A, CSNK1A1, HIRA, FARP2, SHOC2, KDM6B, HSPA8, AKT3, ZNF514, FAM109B, ZBTB4, RUFY2, ARX, SNX5, PTEN, TLN1, KDM5B, SMEK2*
	miR-379-5p	*POU2F1, MGST1, COA1, CCNA2, KIF20A, PRPF40A, MCL1, MAPKAP1, ANPEP, HUWE1, MNT, TUBB, AGO2, PDIA6, TPM3, TAOK1, COL6A1, DHX32, FIS1, MME, NFAT5, RIF1, POLR2A, CASP3, KDM4A, GCN1L1, MGMT, IGF2, COL5A1, RPS16, PABPC1, GDI1, TFPI2, LUM, HSPA8, BCL6, NFE2L1, GHITM, DDX39B, GPI, ZFHX4, CAD, COPA, CREBBP, SERPINE1, AP3M1, GAK, KIAA1279, TMEM165, BAZ2B, RAPH1, SIN3A, HIST1H3B, HIST1H3C*
Protein complex (GO:0043234)	miR-519a-3p	*DNAJA1, APC, DYNC1H1, CACUL1, MED17, UBE3A, CSNK1A1, HIRA, SHOC2, HSPA8, ARCN1, CBX7, CDKN1A, AP2S1, MAP1B*
	miR-379-5p	*KIF20A, MCL1, ITGA5, TUBB, AGO2, COPS2, COL6A1, FIS1, POLR2A, COL5A1, GDI1, LUM, HSPA8, DDX39B, CAD, COPA, LEPR, CREBBP, AP3M1, LMO4, SIN3A, HIST1H3B, HIST1H3C*
Cytosol (GO:0005829)	miR-519a-3p	*DNAJA1, APC, DYNC1H1, TNRC6B, UBE3A, CSNK1A1, FARP2, HSPA8, ARCN1, CDKN1A, PTEN, AP2S1, TLN1, MAP1B*
	miR-379-5p	*MCL1, MAPKAP1, TUBB, AGO2, TPM3, TAOK1, UBE2L6, CASP3, RPS16, EIF4G1, PABPC1, GDI1, HSPA8, GPI, CAD, COPA*
Nucleoplasm (GO:0005654)	miR-519a-3p	*MED17, HIRA, KDM6B, CDKN1A*
	miR-379-5p	*POU2F1, CCNA2, KIF20A, POLR2A, CASP3, MGMT, CREBBP, SIN3A, HIST1H3B, HIST1H3C*
Male pronucleus (GO:0001940)	miR-379-5p	*CCNA2, RIF1*
Prp19 complex (GO:0000974)	miR-519a-3p	*HSPA8*
	miR-379-5p	*POLR2A, HSPA8*
Cytoplasmic side of endoplasmic reticulum membrane (GO:0098554)	miR-519a-3p	*DNAJA1*
Female pronucleus (GO:0001939)	miR-379-5p	*CCNA2, RIF1*
Protein phosphatase type 1 complex (GO:0000164)	miR-519a-3p	*SHOC2*
	miR-379-5p	*PPP1R15B*
**Cellular component (GO:0005575): 113 Target Genes**		

C

GO Category	miRNAs	Target Genes
Enzyme binding (GO:0019899)	miR-519a-3p	*DNAJA1, APC, CACUL1, SHOC2, HSPA8, CDKN1A, ZBTB4, PTEN*
	miR-379-5p	*CCNA2, KIF20A, MAPKAP1, WNK1, POLR2A, KDM4A, HSPA8, CAD, SERPINE1, AP3M1*
Ion binding (GO:0043167)	miR-519a-3p	*DNAJA1, ZNF19, DYNC1H1, TIMP2, KLF10, RASD1, ZXDB, ARL1, PFN2, CSNK1A1, KDM6B, HSPA8, AKT3, ZNF514, ENPP5, CDKN1A, ZBTB4, RUFY2, SNX5, PTEN, KDM5B*
	miR-379-5p	*KIF20A, MAPKAP1, ANPEP, WNK1, ITGA5, TUBB, PAPPA, GALNT11, AGO2, HMOX2, TAOK1, YME1L1, DHX32, MME, POLR2A, KDM4A, COL5A1, HSPA8, BCL6, DDX39B, ZFHX4, CAD, CREBBP, GAK, BAZ2B, LMO4*

Nucleic acid binding transcription factor activity (GO:0001071)	miR-519a-3p	*MYBL1, ZNF19, KLF10, HIRA, ZNF514, KDM5B*
	miR-379-5p	*POU2F1, MNT, NFAT5, BCL6, NFE2L1, CREBBP, LMO4*
Enzyme regulator activity (GO:0030234)	miR-519a-3p	*APC, TIMP2, ARL1, PFN2, SHOC2, CDKN1A*
	miR-379-5p	*WNK1, IGF2, GDI1, TFPI2, SERPINE1*
**Molecular function (GO:0003674): 115 Target Genes**		
